# Efficacy of Vonoprazan in Nonsteroidal Anti-Inflammatory Drug-Induced Ulcer in Terms of Ulcer Recurrence and Gastrointestinal Bleeding: A Systematic Review and Meta-Analysis

**DOI:** 10.1155/grp/5625149

**Published:** 2025-11-13

**Authors:** Sanjay Bandyopadhyay, Shambo Samrat Samajdar, Saibal Das

**Affiliations:** ^1^Department of Gastroenterology, ILS Dumdum Hospital, Kolkata, West Benagal, India; ^2^Department of Clinical and Experimental Pharmacology, Calcutta School of Tropical Medicine, Kolkata, West Bengal, India; ^3^Department of Pharmacology, JMN Medical College, Nadia, West Bengal, India; ^4^Indian Council of Medical Research-Centre for Ageing and Mental Health, Kolkata, West Bengal, India; ^5^Department of Global Public Health, Karolinska Institutet, Stockholm, Sweden

**Keywords:** bleeding, nonsteroidal anti-inflammatory drug (NSAID), proton pump inhibitors (PPIs), ulcer, vonoprazan

## Abstract

**Objective:**

To evaluate the efficacy and safety of vonoprazan therapy as compared to conventional proton pump inhibitors (PPIs) or no vonoprazan for nonsteroidal anti-inflammatory drug (NSAID)-induced ulcers.

**Methods:**

A literature search was conducted across databases (PubMed, Embase, Scopus, Cochrane CENTRAL, and ClinicalTrials.gov). The primary outcome was the risk of ulcer recurrence. Secondary outcomes comprised the risk of gastrointestinal (gastric/duodenal) bleeding; serum gastrin, pepsinogen I, and pepsinogen II levels; and safety. Pooled relative risks (RRs) and mean differences with a 95% confidence interval (CI) were determined, as appropriate, utilizing random effects models.

**Results:**

A total of 744 articles were screened and three of them were included. The overall proportion of ulcer recurrence with vonoprazan therapy was 1.88% (95% CI: 0.98, 3.6) and the overall proportion of gastrointestinal (gastric/duodenal) bleeding with vonoprazan therapy was 1.03 (95% CI: 0.64, 1.64). As compared to PPI (lansoprazole), vonoprazan treatment led to a significant reduction in the risks of ulcer recurrence (RR: 0.55 (95% CI: 0.31, 0.97), *p* = 0.04, *i*^2^ = 0%) and gastrointestinal bleeding (RR: 0.40 (95% CI: 0.16, 0.97), *p* = 0.04, *i*^2^ = 0%). Vonoprazan treatment led to a dose-dependent significant increase in serum gastrin, serum pepsinogen I, and serum pepsinogen II levels. There was no significant difference in the risk of any adverse event (RR: 0.99 (95% CI: 0.94, 1.04), *p* = 0.64, *i*^2^ = 36%) following vonoprazan therapy as compared to lansoprazole. The GRADE of evidence was moderate.

**Conclusion:**

Vonoprazan could significantly reduce the risk of ulcer recurrence and gastrointestinal bleeding as compared to lansoprazole in patients with NSAID-induced ulcers.

## 1. Introduction

Nonsteroidal anti-inflammatory drugs (NSAIDs) are widely prescribed for their analgesic and anti-inflammatory properties, but their use can lead to gastrointestinal complications, notably NSAID-induced ulcers. These ulcers develop due to the inhibition of cyclooxygenase enzymes, which disrupt the protective prostaglandin synthesis in the gastrointestinal mucosa, thereby compromising its integrity [1, 2]. Prolonged NSAID usage increases the risk of ulcer formation, with factors such as dose, duration, and individual susceptibility playing significant roles. NSAID-induced ulcers pose a substantial burden on public health, often resulting in complications such as bleeding, perforation, and obstruction, necessitating hospitalization and even surgical intervention. The elderly, those with a history of peptic ulcers, and concurrent users of corticosteroids or anticoagulants are particularly vulnerable populations [1, 2].

The current recommendations advise patients with NSAID-induced ulcers to begin by ceasing NSAID use [3], yet implementing this approach can pose challenges, mainly when considering the potential recurrence of arthritic pain and its detrimental effects on quality of life [4]. One of the common management strategies for NSAID-induced ulcers involves the use of proton pump inhibitors (PPIs) which help allay the risk of ulcer development by counteracting the reduction in prostaglandin levels [5]. Unlike traditional PPIs, vonoprazan fumarate competitively inhibits H^+^/K^+^-ATPase by binding to the potassium ion site, eliminating the need for activation by gastric acid. [6].

From a clinical point of view, vonoprazan exhibits several benefits compared to PPIs. For example, vonoprazan's activation does not rely on an acidic environment, remaining acid stable [7], which obviates the necessity for an enteric-coated formulation. In contrast to PPIs, which typically take around 3–5 days to achieve maximal inhibition of gastric H^+^/K^+^-ATPase [8], vonoprazan delivers near-maximum inhibitory impact starting from the initial dose and sustains effectiveness for a full 24-h period [9]. Vonoprazan offers robust and prolonged suppression of gastric acid secretion [10, 11]. Numerous investigations have been undertaken to assess the effectiveness and safety of vonoprazan in the context of NSAID-induced ulcers. Therefore, this systematic review and meta-analysis are aimed at comprehensively synthesizing both qualitative and quantitative evidence.

## 2. Methods

### 2.1. Search Strategy and Selection Criteria

In this study, an extensive search was conducted across several prominent databases including PubMed, Embase, Scopus, Cochrane CENTRAL, and ClinicalTrials.gov. The objective was to identify relevant English-language studies published until 31^st^ May 2024, focusing on randomized controlled trials (RCTs) and observational studies. These studies examined the comparative effects of oral vonoprazan versus PPIs in adult patients (≥ 18 years old, any gender) with NSAID-induced gastric or duodenal ulcers. Excluded were studies involving patients without a documented history of NSAID use for these ulcers. The search strategy employed tailored terms specific to each database to ensure comprehensive coverage. We followed the methods of Bandyopadhyay et al. [12]. Two independent reviewers assessed the titles and abstracts of identified studies without language restrictions. Abstracts and, when necessary, full texts were screened using Rayyan software (https://www.rayyan.ai/) to determine eligibility. The authors of relevant articles were contacted via email to obtain missing information. Full-text articles were prioritized, with conference papers, reviews, commentaries, and other nonprimary materials excluded from the analysis. Detailed information on the literature search approach can be found in Table S1. The primary outcome was the risk of ulcer recurrence, while secondary outcomes included assessing the risks of gastrointestinal bleeding and levels of serum gastrin, pepsinogen I, and pepsinogen II, as well as evaluating the safety profile associated with vonoprazan treatment. Ulcer recurrence was variably defined between studies to include endoscopic evidence of mucosal breaks and clinically suspected recurrence on symptomatology with or without follow-up endoscopy. Gastrointestinal bleeding was operationally defined as overt bleeding (e.g., hematemesis and melena) documented by endoscopy, clinical signs, or laboratory evidence of a substantial hemoglobin drop or requirement for medical treatment.

### 2.2. Data Analysis

During the abstract review and data extraction, three authors independently conducted these tasks using a predesigned data extraction spreadsheet. The process emphasized accuracy and reliability, avoiding assumptions or oversimplifications. Selected studies underwent a risk-of-bias assessment using the revised Cochrane Risk-of-Bias 2 tools [13] for RCTs and the Newcastle–Ottawa scale [14] for observational studies. Summary estimates were utilized, and meta-analysis was performed where adequate data existed for predetermined primary and secondary outcomes. Statistical analyses were conducted using RevMan Version 5.3 software. A random-effects model was chosen to enhance the robustness of the model across diverse populations and to address potential outliers. Pooled relative risks (RRs) and mean differences with corresponding 95% confidence intervals (CIs) were calculated as appropriate using random-effects models.

Descriptive statistics were employed when data were insufficient. Attrition rates, including dropouts, loss to follow-up, and withdrawals, were considered in the analysis, which also involved a critical evaluation of missing data and imputation methods [15]. Heterogeneity was assessed using the *χ*^2^ test with *n* − 1 degrees of freedom, a 5% *α* error for statistical significance, and the *I*^2^ test [16], with *I*^2^ values categorized as low (25%), medium (50%), and high (75%). Funnel plots were generated to explore the potential impact of small study effects and publication bias [15, 17]. The GRADE (Grading of Recommendations Assessment, Development, and Evaluation) approach was applied to evaluate the quality of evidence generated in this study [18]. This meta-analysis adheres to the guidelines outlined in the Preferred Reporting Items for Systematic Reviews and Meta-Analyses (PRISMA). The research protocol was registered in the International Prospective Register of Systematic Reviews (PROSPERO ID: CRD42024544680).

## 3. Results

**Figure 1:**
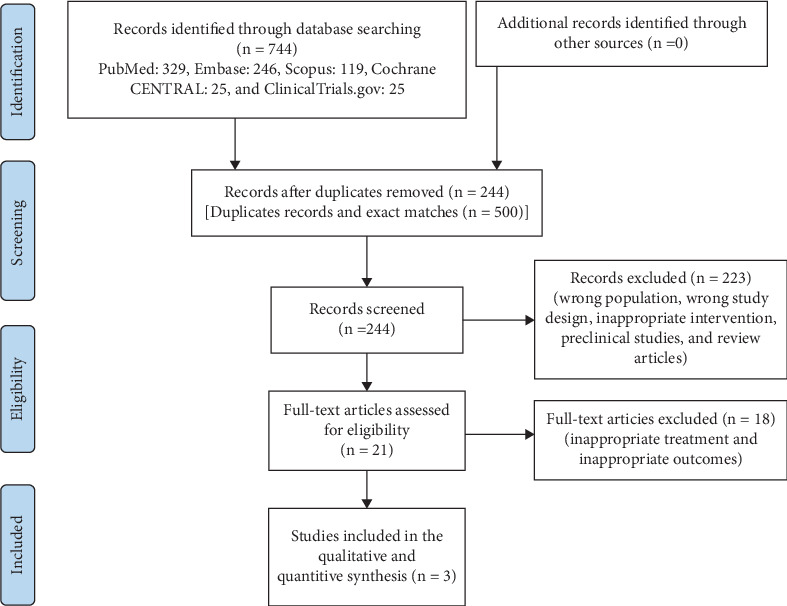
Study flow chart.

Out of a total of 744 articles screened, three [19, 20] met the inclusion criteria and were included in the final analysis (Figure 1). Among these, there were two RCTs [19, 20] and one observational study [21]. The study characteristics are tabulated in Table 1. Two studies [19, 21] included patients with chronic painful conditions developing NSAID-induced ulcers while one [21] included patients with ischemic heart disease developing low-dose aspirin-induced ulcers. All the studies exhibited a low risk of bias (Figure S1). Across various studies, the dose of vonoprazan ranged from 10 to 20 mg, administered for durations spanning 6–12 months. Two studies [19, 20] had an active comparator arm (lansoprazole 15 mg). The *Helicobacter pylori* status was comparable across both groups. Recurrence of ulcers in two studies was measured by planned endoscopy at follow-up [19, 20]. In one study, it was ascertained by clinical assessment, patient complaints, and documentation of medical records [21]. Gastrointestinal bleeding episodes were identified by clinical symptoms (melena and hematemesis), hemoglobin trend, and requirement of intervention or hospitalization in all three studies.

**Figure 2:**
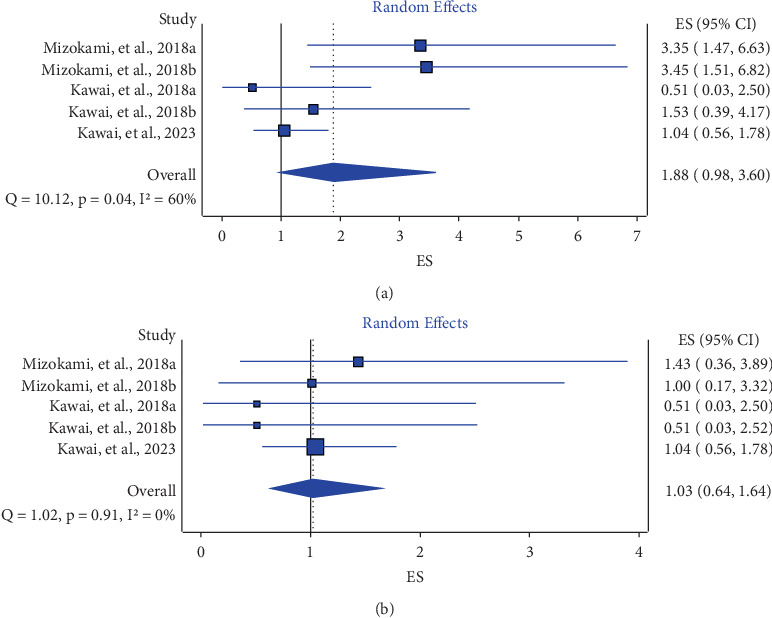
The overall proportion of ulcer recurrence with vonoprazan therapy (a) and the overall proportion of gastrointestinal (gastric/duodenal) bleeding with vonoprazan therapy (b) (the suffixes “a” and “b” after the publication year denote vonoprazan doses of 10 and 20 mg, respectively). For the study Kawai et al. [20], the event rate in the vonoprazan arm was approximated to 1 for calculation.

The overall proportion of ulcer recurrence with vonoprazan therapy was 1.88% (95% CI: 0.98, 3.6) (*i*^2^ = 60%) (Figure 2a), and the overall proportion of gastrointestinal (gastric/duodenal) bleeding with vonoprazan therapy was 1.03 (95% CI: 0.64, 1.64) (*i*^2^ = 0%) (Figure 2b). As compared to PPI (lansoprazole), vonoprazan treatment led to a significant reduction in the risks of ulcer recurrence (RR: 0.55 (95% CI: 0.31, 0.97), *p* = 0.04, *i*^2^ = 0%) (moderate GRADE evidence) (Figure S2) and gastrointestinal bleeding (RR: 0.40 (95% CI: 0.16, 0.97), *p* = 0.04, *i*^2^ = 0%) regardless of patient *Helicobacter pylori* status (moderate GRADE evidence) (Figure S3).

In the study by Mizokami et al. [19] and Kawai et al. [20], the noninferiority of vonoprazan 10 and 20 mg to lansoprazole 15 mg was verified for recurrent ulcer within 24 weeks of treatment and the beneficial effect of vonoprazan persisted in the extension phase of the study (2 and 1.5 years, respectively). The Kaplan–Meier cumulative incidence rates of ulcer recurrence and gastrointestinal bleeding occurrence were similar or lower in both vonoprazan groups compared to the lansoprazole 15 mg group. In the study by Kawai et al. [20], the bleeding incidence was higher among patients receiving oral antithrombotic drugs. In the study by Kawai et al. [21], the incidence of ulcer recurrence was twofold or more in patients with self-reported *Helicobacter pylori* infection and in those who had low body mass index (< 18.5 kg/m^2^).

As compared to PPI or no vonoprazan therapy, vonoprazan treatment led to a dose-dependent significant increase in the serum gastrin (mean difference: 382.61 pg/mL (95% CI: 334.40, 430.83), *p* < 0.001, *i*^2^ = 0%) (moderate GRADE evidence) (Figure S4), serum pepsinogen I (mean difference: 56.74 ng/mL (95% CI: 39.97, 73.52), *p* < 0.001, *i*^2^ = 36%) (moderate GRADE evidence) (Figure S5), and serum pepsinogen II (mean difference: 7.65 ng/mL (95% CI: 4.47, 10.83), *p* < 0.001, *i*^2^ = 66%) (moderate GRADE evidence) (Figure S6) levels. In the study by Kawai et al. [21], the mean serum gastrin level was 365.6 ± 395.7 pg/mL at the start of vonoprazan therapy and was 880.8 ± 551.5 pg/mL at the end of the study.

Regarding safety, methods of collecting adverse events differed among studies. The two RCTs utilized scheduled and structured adverse event monitoring methods, whereas the observational study utilized pharmacovigilance data with a less formal adverse event capture process (Table S3). There was no significant difference in the risk of any adverse event (RR: 0.99 (95% CI: 0.94, 1.04), *p* = 0.64, *i*^2^ = 36%) (moderate GRADE evidence) (Figure S7) or serious adverse event (RR: 1.15 (95% CI: 0.90, 1.48), *p* = 0.26, *i*^2^ = 0%) (moderate GRADE evidence) (Figure S8) following vonoprazan therapy as compared to lansoprazole. The overall proportion of adverse events was 39.43% (95% CI: 22.96, 67.72) (*i*^2^ = 98%) (Figure S9). The common adverse events reported with vonoprazan therapy were nasopharyngitis, falls, gastrointestinal disorders, nervous system disorders, and hepatobiliary system disorders, and they were all mild in nature. In the study by Kawai et al. [21], the incidence of AE was numerically higher in female patients than in male patients, in younger patients than in older patients, and in those with comorbidities.

## 4. Discussion

In this systematic review and meta-analysis encompassing 2531 patients, the results suggest that vonoprazan treatment may offer substantial advantages for individuals with NSAID-induced ulcers. Furthermore, the treatment was deemed generally safe and on par with PPIs. Several guidelines advocate for the concurrent utilization of PPIs or prostaglandin analogs alongside NSAIDs to prevent NSAID-induced ulcers. [21] Furthermore, PPIs may also lead to the development of adverse effects [22]. Limited research has directly juxtaposed these agents; nevertheless, a meta-analysis unveiled that while PPIs, double-dose histamine receptor antagonists, and prostaglandin analogs are efficacious in secondary ulcer prevention, they may not offer complete effectiveness [23]. Hence, PPIs, which boast higher efficacy and fewer adverse events, might be regarded as the optimal choice for preventing secondary ulcers [23]. The outcomes of this study align with earlier research that explored the efficacy of PPIs in preventing secondary ulcers [24]. Moreover, they illustrate that vonoprazan exhibits efficacy on par with lansoprazole. The outcomes obtained with vonoprazan are favorable when compared to previous findings for lansoprazole 15 mg [25, 26] and are consistent with those reported for esomeprazole 20 mg [27] and rabeprazole 5 and 10 mg [28] in studies conducted with similar designs and patient cohorts.

The percentage of patients experiencing recurrent ulcers and gastrointestinal bleeding was lower with vonoprazan compared to lansoprazole, although these variances did not reach statistical significance. Thus, vonoprazan presents itself as a potentially valuable clinical alternative to PPIs, particularly for individuals with high-risk factors. Given that not all NSAID users develop peptic ulcers during treatment, this study specifically recruited individuals at elevated risk, including those with a history of endoscopically confirmed peptic ulcers and a need for continuous, long-term NSAID therapy (inclusive of selective cyclooxygenase-2 inhibitors) for pain management purposes. Additionally, patients with other risk factors were incorporated, including advanced age, *Helicobacter pylori* infection, smoking, alcohol consumption, and concurrent use of anticoagulant therapy. The serum gastrin and pepsinogen I and II levels were consistently higher in the vonoprazan treatment groups as compared with the lansoprazole treatment group, and the rises were dose-dependent. Nevertheless, the alterations were deemed not clinically significant, and no patients withdrew from either treatment cohort. The precise mechanism behind this elevation in these parameters remains incompletely understood and could be attributed to vonoprazan's robust gastric acid antisecretory effect; nonetheless, similar effects have been observed with other PPIs [29].

PPIs act as inactive compounds that need acid activation before they can covalently bind to the gastric H^+^/K^+^-ATPase via a disulfide linkage. PPIs are predominantly metabolized by cytochrome P450 (CYP) enzymes, particularly CYP2C19 and CYP3A4, which are crucial in their metabolic inactivation [30]. Although PPIs have shown efficacy, they are associated with specific drawbacks. These drawbacks encompass delayed onset of action (usually 3–5 days), limited bioavailability, potential for nocturnal acid breakthrough, susceptibility to drug interactions, and the necessity for precise timing in relation to meals to achieve optimal efficacy [30]. Moreover, PPIs exhibit notable interindividual variability in pharmacodynamics and clinical efficacy, which is linked to variations in cytochrome polymorphisms. Furthermore, increasing attention is being given to the escalating issue of antibiotic resistance associated with treatment protocols utilizing PPIs to eliminate concurrent *Helicobacter pylori* infection [30].

Recent systematic reviews and meta-analyses have indicated an association between PPI usage and heightened fracture risk [31] as well as an increased incidence of gastroenteritis infections caused by *Clostridium difficile* [32]. Prolonged utilization of PPIs has also been linked to a potentially heightened risk of gastrointestinal cancer, which may be connected to hypergastrinemia, although the correlation remains uncertain [33]. Vonoprazan was well tolerated in the study. The higher proportion of adverse events in the two RCTs [20, 21] as compared to the observational study (postmarketing surveillance study) could be attributed to the differences in the definition of adverse event and the stringency in monitoring the same. Throughout extended treatment periods, the safety profiles of the 10 and 20 mg doses resembled that of lansoprazole 15 mg. According to the study conducted by Kawai et al. in 2022, over 85% of patients managed to sustain vonoprazan treatment for at least 6 months, with a low incidence of adverse events (0.71%) and no emergence of new safety issues. Similar to the findings of the study by Kawai et al., the results of a long-term post-marketing surveillance of the safety and effectiveness of vonoprazan-based *Helicobacter pylori* eradication therapy [34] showed that the incidence of adverse events was significantly higher in female patients than in male patients. The characteristics shown to be associated with ulcer recurrence were consistent with the known or potential factors associated with recurrent ulcer and gastrointestinal bleeding [35]. A recent systematic review and meta-analysis have further affirmed the well-tolerated nature of vonoprazan, indicating comparable safety to PPIs. The safety profile of vonoprazan may primarily hinge on its indications and duration [36].

Potassium-competitive acid blockers (P-CABs) represent a novel and diverse class of drugs that competitively inhibit the potassium-binding site of the gastric H^+^/K^+^-ATPase. After entering the systemic circulation, P-CABs gather in the canalicular membrane of parietal cells, where they undergo the addition of a proton to the molecule in environments that are strongly acidic. Importantly, P-CABs distinguish themselves from PPIs by being acid-stable and not requiring enteric-coated formulations. Additionally, P-CABs exhibit a faster onset of acid suppression and an increase in intragastric pH compared to PPIs. This is because they rapidly reach peak plasma concentrations after oral intake, allowing them to inhibit the H^+^/K^+^-ATPase without requiring proton pump activation [33]. P-CABs like vonoprazan possess the unique capability to achieve full anti-secretory effects with the initial dose and maintain more consistent control of gastric pH compared to PPIs. Vonoprazan is currently the only P-CAB approved for clinical use. Its development focused on overcoming the shortcomings associated with PPIs, such as their brief duration of action, insufficient acid suppression, delayed onset of effects, and vulnerability to metabolic variability [29, 37].

Prevention of recurrence of NSAID-induced ulcers and low-dose aspirin-induced ulcers is indeed different clinical situations due to the varying mechanisms of action and risk profiles of these medications. NSAID-induced ulcers typically result from the inhibition of Cyclooxygenase 1 and 2 enzymes, which reduces protective prostaglandins in the gastric mucosa, necessitating strategies that focus on more potent acid suppression and mucosal protection. In contrast, low-dose aspirin-induced ulcers, while also involving Cyclooxygenase 1 inhibition, primarily present a risk due to the continuous antiplatelet effect, often requiring careful balancing of gastrointestinal protection with the cardiovascular benefits of aspirin, making the choice of protective agents, such as PPIs or vonoprazan, and their dosage particularly critical in preventing ulcer recurrence. Thus, while both conditions require preventative measures, the therapeutic approach must be tailored to address the specific risks and needs associated with each situation.

Our group earlier demonstrated the beneficial effects of vonoprazan in nonerosive gastroesophageal reflux disease [12]. The beneficial effects of vonoprazan in NSAID-induced ulcers were demonstrated in an earlier case report [38]. Previous studies involving patients with peptic ulcers, including those with NSAID-induced ulcers, have also confirmed the beneficial effects of vonoprazan [39–42]. It was also observed that there were no clinically meaningful drug-drug interactions and vonoprazan was well tolerated when administered with NSAIDs. This study offers a novel contribution by focusing on the long-term safety and efficacy of vonoprazan, particularly in high-risk populations with unique ulcerogenic factors, such as low body mass index and *Helicobacter pylori* status. Additionally, our research provides a more detailed comparison of vonoprazan with various PPIs across different dosages and treatment durations, offering new insights into vonoprazan's superior and consistent acid suppression capabilities [43, 44].

The studies incorporated into the analysis present various limitations. Primarily, the limited number of studies and small sample sizes could affect the reliability of the findings, and the outcomes might evolve with the emergence of more comprehensive studies. Additionally, the absence of evaluation of endoscopic or histological parameters is a limitation, as it could provide a more accurate assessment of NSAID-induced ulcers. Throughout the review process, certain outcomes of interest encountered limited data availability, resulting in their exclusion or inability to be incorporated into the meta-analysis. Significant heterogeneity in certain outcomes represents another limitation, indicating that the results may not be consistent across different studies or patient groups. This variance can be ascribed to several factors, including differences in the study population, such as age, comorbidities, and genetic predispositions; the duration and severity of ulcers among participants, which may affect how they respond to treatment; and variations in the dose and duration of vonoprazan therapy, as different treatment protocols and dosing regimens could lead to varying levels of efficacy and safety across the studies. The definition of ulcer recurrence, the primary outcome of this study, differed across the included studies. This heterogeneity complicates the interpretation of the overall effectiveness and generalizability of vonoprazan for treating NSAID-induced ulcers. Also, there was a risk of multiple comparisons (vonoprazan 10 and 20 mg) with the same comparator (lansoprazole 15 mg) [20, 21]. The overall quality of evidence, as assessed by the GRADE, was moderate for some outcomes, suggesting the need for further research with larger sample sizes and a more comprehensive evaluation of histological parameters to enhance the reliability and generalizability of the findings.

## 5. Conclusion

The findings from this study suggest that vonoprazan could be a valuable alternative to PPIs in the treatment of NSAID-induced ulcers, offering comparable efficacy with potentially faster and more consistent acid suppression. This could enhance treatment outcomes, particularly for patients with high-risk factors, by providing a more rapid onset of ulcer healing and reducing the likelihood of recurrent ulcers and gastrointestinal bleeding. Additionally, the favorable safety profile of vonoprazan, even during long-term use, may allow for broader application in managing NSAID-induced gastropathy, particularly in populations where PPI use is complicated by drug interactions or metabolic variability.

## Figures and Tables

**Table 1:** Characteristics of the included studies.

**Author, year, country**	**Type of study**	**Age (years)**	**n** **(male: female)**	**Comorbidities**	**Nonsteroidal anti-inflammatory drugs used**	**Concomitant medications used**	**Interventional arm**	**Comparator arm**
Mizokami, et al., 2018, Japan [19]	Randomized controlled trial	• Arm 1: 65 ± 11.6 • Arm 2: 64.9 ± 11.4 • Arm 3: 65.2 ± 10.4	• Arm 1: 218 (89: 129) • Arm 2: 212 (92: 120) • Arm 3: 212 (75: 137)	Rheumatoid arthritis, osteoarthritis, and others	Selective cyclooxygenase-2 inhibitors (celecoxib and rofecoxib with paracetamol) and others	Biologicals, disease-modified antirheumatic drugs, and supplements	• Arm 1: vonoprazan 10 mg once daily for 24 weeks (extension phase for 2 years) • Arm 2: vonoprazan 20 mg once daily for 24 weeks (extension phase for 2 years)	• Arm 3: lansoprazole 15 mg for 24 weeks (extension phase for 2 years)

Kawai, et al., 2018, Japan [20]	Randomized controlled trial	• Arm 1: 68.9 ± 8 • Arm 2: 69.1 ± 7.2 • Arm 3: 68.3 ± 9.1	• Arm 1: 202 (166: 36) • Arm 2: 202 (163: 39) • Arm 3: 217 (178: 39)	Ischemic heart disease, ischemic cerebrovascular disorder, and others	Aspirin	ACE inhibitors, ARBs, beta-blockers, calcium channel blockers, antiplatelets, and statins	• Arm 1: Vonoprazan 10 mg once daily for 24 weeks (extension phase for 1.5 years) • Arm 2: vonoprazan 20 mg once daily for 24 weeks (extension phase for 1.5 years)	• Arm 3: Lansoprazole 15 mg for 24 weeks (extension phase for 1.5 years)

Kawai, et al., 2023, Japan [21]	Observational study	70.9 ± 13.5	1268 (429: 839)	Rheumatoid arthritis, osteoarthritis, and others	Selective cyclooxygenase-2 inhibitors (celecoxib and rofecoxib with paracetamol) and others	Biologicals, disease-modified antirheumatic drugs, and supplements	12-month prospective observational study	—

## Data Availability

The original contributions presented in the study are included in the main article/supporting information. Further inquiries can be directed to the corresponding author.

## Supporting Information

Supporting Information Additional supporting information can be found online in the Supporting Information section. Table S1: Literature search strategy. The search strategy focused on the NSAID-induced peptic ulcer studies using broad MeSH terms within PubMed and Embase. For the intervention, vonoprazan and related variations were sought on PubMed, Embase, and ClinicalTrials.gov. The aim was to retrieve relevant research and clinical trials of the efficacy of vonoprazan in NSAID-induced ulcers. Table S2: It represents the GRADE evidence quality assessments for outcomes of studies in pooled analyses. GRADE evidence quality assessments were conducted for the outcomes of ulcer recurrence, gastrointestinal bleeding, and serum levels of gastrin, pepsinogen I, and pepsinogen II, based on data from RCTs and observational studies. All outcomes had a grade of moderate certainty and critical importance. Table S3. Figure S1: Risk of bias assessments of the included studies. (a) Both Mizokami et al. [19] and Kawai et al. [20] had low risk across all Cochrane Risk-of-Bias 2 domains. (b) Kawai et al. [21] scored 7/9 on the Newcastle–Ottawa scale, indicating that the study was of high methodological quality in terms of selection, comparability, and outcomes. Figure S2: Comparison of vonoprazan versus lansoprazole for ulcer recurrence vonoprazan 10 mg doses are represented by suffix “a” and 20 mg represented by suffix “b” were compared against lansoprazole. Figure S3: Comparison of vonoprazan with lansoprazole in reducing gastrointestinal bleeding (gastric/duodenal). The “a” and “b” suffixes refer to the 10 and 20 mg vonoprazan, respectively. In Kawai et al.'s [20] study, the event rate for vonoprazan was taken as 1 for the purpose of calculation. Figure S4: Effect of vonoprazan versus lansoprazole on serum gastrin levels. Suffixes “a” and “b” refer to a dose of 10 and 20 mg of vonoprazan, respectively. Figure S5: Time course of serum pepsinogen I levels treated by vonoprazan compared with lansoprazole. Suffixes “a” and “b” mean 10 and 20 mg of vonoprazan, respectively. Figure S6: Comparison of the effect of vonoprazan and lansoprazole on serum pepsinogen II levels. Suffixes “a” and “b” are for vonoprazan at doses of 10 and 20 mg, respectively. Figure S7: Comparison of vonoprazan with lansoprazole in the incidence of adverse events. “a” and “b” represent the 10 and 20 mg dosage of vonoprazan, respectively. Figure S8: Effect of vonoprazanas compared with lansoprazole on serious adverse events (the suffixes “a” and “b” after the publication year denote vonoprazan doses of 10 and 20 mg, respectively). Figure S9: Overview of the proportion of adverse events with vonoprazan. The suffixes “a” and “b” refer to vonoprazan 10 and 20 mg, respectively.
