# Supplementary material for: Efficacy of Vonoprazan in Nonsteroidal Anti-Inflammatory Drug-Induced Ulcer in Terms of Ulcer Recurrence and Gastrointestinal Bleeding: A Systematic Review and Meta-Analysis
Source: Gastroenterol Res Pract. 2025 Nov 13;2025:5625149. doi: 10.1155/grp/5625149 (PMC12634168; doi:10.1155/grp/5625149)
Supplement: Supporting Information — Additional supporting information can be found online in the Supporting Information section. Table S1: Literature search strategy. The search strategy focused on the NSAID-induced peptic ulcer studies using broad MeSH terms within PubMed and Embase. For the intervention, vonoprazan and related variations were sought on PubMed, Embase, and ClinicalTrials.gov. The aim was to retrieve relevant research and clinical trials of the efficacy of vonoprazan in NSAID-induced ulcers. Table S2: It represents the GRADE evidence quality assessments for outcomes of studies in pooled analyses. GRADE evidence quality assessments were conducted for the outcomes of ulcer recurrence, gastrointestinal bleeding, and serum levels of gastrin, pepsinogen I, and pepsinogen II, based on data from RCTs and observational studies. All outcomes had a grade of moderate certainty and critical importance. Table S3. Figure S1: Risk of bias assessments of the included studies. (a) Both Mizokami et al. [19] and Kawai et al. [20] had low risk across all Cochrane Risk-of-Bias 2 domains. (b) Kawai et al. [21] scored 7/9 on the Newcastle–Ottawa scale, indicating that the study was of high methodological quality in terms of selection, comparability, and outcomes. Figure S2: Comparison of vonoprazan versus lansoprazole for ulcer recurrence vonoprazan 10 mg doses are represented by suffix “a” and 20 mg represented by suffix “b” were compared against lansoprazole. Figure S3: Comparison of vonoprazan with lansoprazole in reducing gastrointestinal bleeding (gastric/duodenal). The “a” and “b” suffixes refer to the 10 and 20 mg vonoprazan, respectively. In Kawai et al.'s [20] study, the event rate for vonoprazan was taken as 1 for the purpose of calculation. Figure S4: Effect of vonoprazan versus lansoprazole on serum gastrin levels. Suffixes “a” and “b” refer to a dose of 10 and 20 mg of vonoprazan, respectively. Figure S5: Time course of serum pepsinogen I levels treated by vonoprazan compared with lansoprazole. [file 5625149.f1.docx]

**Supplementary Materials**

**Table s1.** Literature search strategy.

| Patients | PubMed, Embase | Peptic Ulcers[MeSH] OR Ulcer, Peptic[MeSH] OR Ulcers, Peptic[MeSH] OR Gastroduodenal Ulcer[MeSH] OR Gastroduodenal Ulcers[MeSH] OR Ulcer, Gastroduodenal[MeSH] OR Ulcers, Gastroduodenal[MeSH] OR Marginal Ulcer[MeSH] OR Marginal Ulcers[MeSH] OR Ulcer, Marginal[MeSH] OR Ulcers, Marginal[MeSH] OR Ulcer, Peptic[MeSH] OR NSAID[MeSH] OR Nonsteroidal Anti-Inflammatory Agent[MeSH] OR Agent, Nonsteroidal Anti-Inflammatory[MeSH] OR Anti-Inflammatory Agent, Nonsteroidal[MeSH] OR Nonsteroidal Anti Inflammatory Agent[MeSH] OR NSAIDs[MeSH] OR Antiinflammatory Agents, Non Steroidal[MeSH] OR Antiinflammatory Agents, Nonsteroidal[MeSH] OR Nonsteroidal Antiinflammatory Agents[MeSH] OR Non-Steroidal Anti-Inflammatory Agents[MeSH] OR Non Steroidal Anti Inflammatory Agents[MeSH] OR Nonsteroidal Anti-Inflammatory Agents[MeSH] OR Nonsteroidal Anti Inflammatory Agents[MeSH] OR Non-Steroidal Anti-Inflammatory Agent[MeSH] OR Agent, Non-Steroidal Anti-Inflammatory[MeSH] OR Anti-Inflammatory Agent, Non-Steroidal[MeSH] OR Non Steroidal Anti Inflammatory Agent[MeSH] OR Anti Inflammatory Agents, Nonsteroidal[MeSH] OR Analgesics, Anti-Inflammatory[MeSH] OR Anti-Inflammatory Analgesics[MeSH] OR Aspirin-Like Agents[MeSH] OR Aspirin Like Agents[MeSH] OR Aspirin-Like Agent[MeSH] OR Agent, Aspirin-Like[MeSH] OR Aspirin Like Agent |
| --- | --- | --- |
|  | Clinical[Trials.gov](http://trial.gov/) | Vonoprazan AND non-steroidal anti-inflammatory agents-induced ulcer |
| Intervention | PubMed, Embase | Vonoprazan[MeSH] OR TAK 438[MeSH] OR TAK438[MeSH] OR TAK-438[MeSH] |
|  | Clinical[Trials.gov](http://trial.gov/) | Vonoprazan AND non-steroidal anti-inflammatory agents-induced ulcer |

**Table s2.** GRADE tables representing the quality of generated evidence for the outcomes of the studies for which pooled analyses were performed.

| **Certainty assessment** | | | | | | | **№ of patients** | | **Effect** | | **Certainty** | **Importance** |  |
| --- | --- | --- | --- | --- | --- | --- | --- | --- | --- | --- | --- | --- | --- |
| **№ of studies** | **Study design** | **Risk of bias** | **Inconsistency** | **Indirectness** | **Imprecision** | **Other considerations** | **[intervention]** | **[comparison]** | **Relative (95% CI)** | **Absolute (95% CI)** |  |  |  |
| Ulcer recurrence | | | | | | | | | | | | | |
| 3 | RCTs and observational study | not serious | not serious | not serious | not serious | none | 18/805 | 34/824 | - | RR 0.55 less (0.31 less to 0.97 less) | ⨁⨁⨁ Moderate | CRITICAL |  |
| Gastrointestinal (gastric/duodenal) bleeding | | | | | | | | | | | | | |
| 3 | RCTs and observational study | not serious | not serious | not serious | not serious | none | 7/802 | 20/826 | - | RR 0.40 less (0.16 less to 0.97 less) | ⨁⨁⨁ Moderate | CRITICAL |  |
| Serum gastrin level | | | | | | | | | | | | | |
| 3 | RCTs and observational study | not serious | not serious | not serious | not serious | none | 779 | 776 | - | RR 382.61 more (334.40 more to 430.83 more) | ⨁⨁⨁ Moderate | CRITICAL |  |
| Serum pepsinogen I level | | | | | | | | | | | | | |
| 3 | RCTs and observational study | not serious | not serious | not serious | not serious | none | 779 | 776 |  | MD 56.74 more (39.97 more to 73.52 more) | ⨁⨁⨁ Moderate | CRITICAL |  |
| Serum pepsinogen II level | | | | | | | | | | | | | |
| 3 | RCTs and observational study | not serious | not serious | not serious | not serious | none | 779 | 776 |  | MD 7.65 more (4.47 more to 10.83 more) | ⨁⨁⨁ Moderate | CRITICAL |  |

CI: confidence interval; MD: mean difference; RCTs: randomized control trials.

**Table s3.** Adverse event collection methods in each included study.

| **Study** | **AE collection method** | **Frequency of monitoring** | **Reporting system used** |
| --- | --- | --- | --- |
| Mizokami et al., 2018 | Structured case report forms and scheduled visits | Every 4-6 weeks | Trial-specific safety protocol |
| Kawai et al., 2018 | Structured case report forms and follow-up visits | Every 4-6 weeks | Trial-specific safety protocol |
| Kawai et al., 2023 | Clinician reports and patient-reported outcomes | Routine clinical practice | Post-marketing surveillance |

| **Author, year** | **Bias arising from the randomization process** | **Bias due to deviations from intended interventions** | **Bias due to missing outcome data** | **Bias due to measurement of the outcome** | **Bias in selection of the reported result** | **Overall bias** |
| --- | --- | --- | --- | --- | --- | --- |
| Mizokami, et al., 2018 | Low | Low | Low | Low | Low | Low |
| Kawai, et al., 2018 | Low | Low | Low | Low | Low | Low |

**A**

| **Studies** | **Selection** | **Comparability** | **Outcome** | **Total score** |
| --- | --- | --- | --- | --- |
| Kawai, et al., 2023 | 4/4 | 0/2 | 3/3 | 7/9 |

**B**

**Figure s1.** Results of the risk of bias assessment of the included studies using the revised Cochrane risk-of-bias 2 tool (A) and Newcastle-Ottawa scale (B).


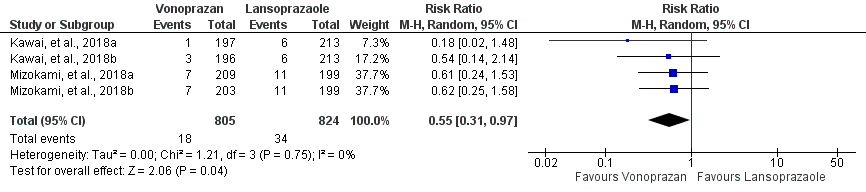


**Figure s2.** Effect of vonoprazan as compared to lansoprazole in causing ulcer recurrence (the suffices ‘a’ and ‘b’ after the publication year denote vonoprazan dose of 10 and 20 mg, respectively).


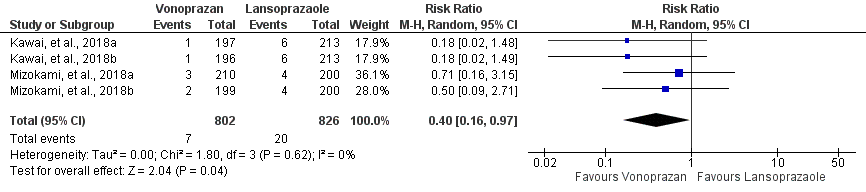


**Figure s3.** Effect of vonoprazan as compared to lansoprazole in causing gastrointestinal (gastric/duodenal) bleeding (the suffices ‘a’ and ‘b’ after the publication year denote vonoprazan dose of 10 and 20 mg, respectively). For the study Kawai, et al., 2018, the event rate in the vonoprazan arm was approximated to 1 for calculation.


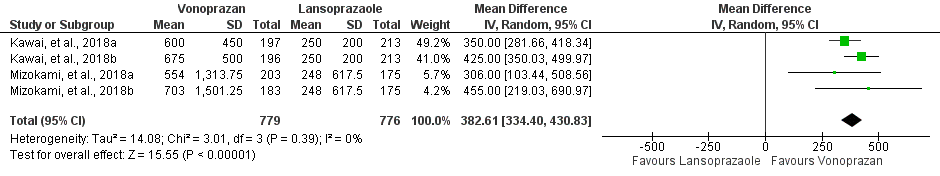


**Figure s4.** Effect of vonoprazan as compared to lansoprazole on serum gastrin level (the suffices ‘a’ and ‘b’ after the publication year denote vonoprazan dose of 10 and 20 mg, respectively).


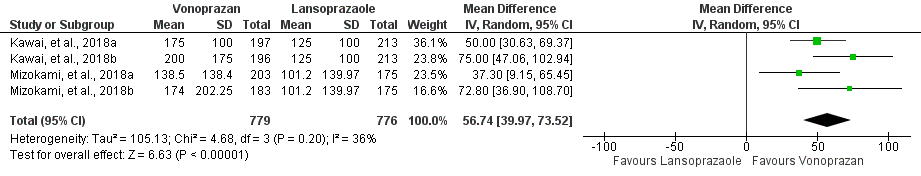


**Figure s5.** Effect of vonoprazan as compared to lansoprazole on serum pepsinogen I level (the suffices ‘a’ and ‘b’ after the publication year denote vonoprazan dose of 10 and 20 mg, respectively).


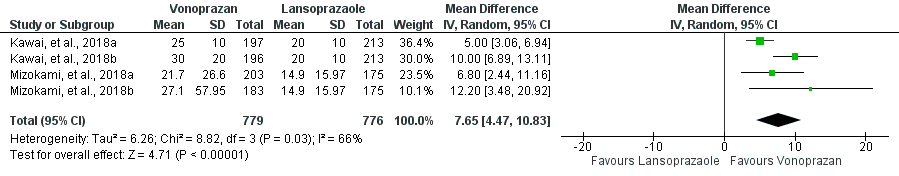


**Figure s6.** Effect of vonoprazan as compared to lansoprazole on serum pepsinogen II level (the suffices ‘a’ and ‘b’ after the publication year denote vonoprazan dose of 10 and 20 mg, respectively).


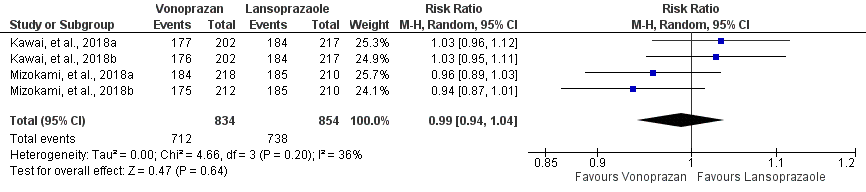


**Figure s7.** Effect of vonoprazan as compared to lansoprazole in causing any adverse event (the suffices ‘a’ and ‘b’ after the publication year denote vonoprazan dose of 10 and 20 mg, respectively).


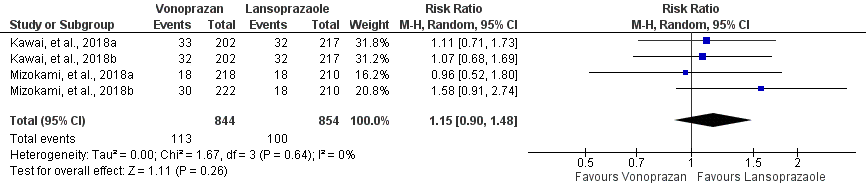


**Figure s8.** Effect of vonoprazan as compared to lansoprazole in causing serious adverse event (the suffices ‘a’ and ‘b’ after the publication year denote vonoprazan dose of 10 and 20 mg, respectively).

**Figure s9.** The overall proportion of adverse events with vonoprazan (the suffices ‘a’ and ‘b’ after the publication year denote vonoprazan dose of 10 and 20 mg, respectively).
